# Occupational injuries in orthopedic and trauma surgeons in Austria

**DOI:** 10.1007/s00402-024-05200-0

**Published:** 2024-01-24

**Authors:** Clemens Clar, Amir Koutp, Andreas Leithner, Lukas Leitner, Paul Puchwein, Ines Vielgut, Patrick Sadoghi

**Affiliations:** Department of Orthopaedics and Trauma, Medical University of Graz, Auenbruggerplatz 5, 8036 Graz, Austria

**Keywords:** Occupational injuries, Orthopedic surgery, Survey, Austria

## Abstract

**Background:**

The aim of this study was to investigate the frequency and type of injuries during the career of orthopedic and trauma surgeons in Austria. The hypothesis was that the percentage of occupational injuries among orthopedic and trauma surgeons aligns with the incidence reported in the United States, thus indicating the need for a workplace prevention program.

**Methods:**

A web-based survey was created to collect all necessary data and was sent to all orthopedic and trauma surgeons in Austria via e-mail. Relevant parameters included the surgeons’ age, work experience, severity of pain, type of injury, and current pain. Descriptive and explorative statistical analysis was performed.

**Results:**

A web-based survey was sent to 1122 board-certified orthopedic surgeons and residents in Austria via e-mail. In total, the response rate was 135 (12%). Seventy-two surgeons (54%) had suffered from one or more occupational injuries during their career. We detected a significant raise of occupational injuries related to the work life duration in which operations were performed and the prevalence of injuries. Most injuries of surgeons were reported between 21 and 30 years of their professional life. According to the frequency at different locations, the distribution in descending order was 25% with injuries of the hand, 22% of finger(s), 12% of the foot, 10% of the spine, 2% of the neck, 3% of the head, and 2% of the abdomen. A causality of incapacity to work through injuries at the workplace was given as 4%. Four percent stated a sick leave of at least 3 weeks. In 7% of the facilities, there was no optimization of preventive measures following an occupational injury. We found no correlation of injuries and resident status.

**Conclusion:**

Orthopedic surgeons in Austria show a high incidence of occupational injuries in line with the findings of colleagues from the United States. The impact on the health system consists of absenteeism in the workplace of highly specialized health service providers as well as the incapacity to work of a high quality, highly trained workforce of at least 4%. With more preventive measures and more attention and care in the rehabilitation phase after such injuries, a positive effect could be achieved. We believe that residents should be specifically trained on how to avoid such injuries.

## Introduction

Occupational injuries among orthopedic and trauma surgeons is a growing problem in the global healthcare system [1, 2]. Since surgeons are often exposed to risk factors in their professional life such as repetitive motions, long hours of standing or working with sharp and electrical instruments, they are at risk of various sharp or blunt injuries, electrical and needle stick injuries, and cuts [3–6]. Although this is a known issue, the healthcare system has paid little attention to it so far [3]. In Austria, a total of 91,181 work accidents were reported in 2021, which corresponds to an incidence of 1018/100000 [7]. Compared to the numbers of the previous year, an increase in occupational accidents was reported, rising from 82,910 to 91,181 in total, which makes this issue even more pressing [7, 8]. Depending on the risk factor in the workplace, the probability of suffering an injury at least once in one’s career can be as high as 79% in Austria [7]. Due to the aforementioned factors, orthopedic and trauma surgeons are considered a risk group and should, therefore, be protected by preventive measures in the workplace, as suggested from various authors [5, 6, 9]. Furthermore, it has been found that exposing orthopedic and trauma surgeons to this risk also negatively impacts their mental health [10]. Overall, prioritizing the health and well-being of surgeons is critical, as it directly affects their capacity to deliver high-quality patient care and burdens costs for the health care system on the long term [11, 12]. In light of the shortage of doctors in Austria and worldwide, it is essential to ensure that orthopedic and trauma surgeons are provided with a safe working environment to maintain the attractiveness of the specialty.

The aim of this study was to retrospectively capture the number of occupational injuries among orthopedic and trauma surgeons in Austria, identify factors that negatively influence the risk of injuries, and illustrate the impact of occupational injuries on the Austrian healthcare system.

The hypothesis was that the percentage of occupational injuries among orthopedic and trauma surgeons aligns with the incidence reported in the United States [13], thus indicating the need for a workplace prevention program. Furthermore, it is suggested that the number of work absences significantly diminishes the overall performance within healthcare facilities, in terms of sick leave days.

## Materials and methods

A web-based questionnaire was created according to the recommendations of the American Academy of Orthopedic Surgeons and subsequently distributed to all members of the Austrian Society for Orthopedics (ÖGO) via e-mail on January, 18th 2022. The web-based questionnaire consisted of a total of 15 questions. It included inquiries about the workplace, distinguishing between university hospitals, central hospitals, peripheral hospitals, private practices, or other categories. Furthermore, it collected data on specialization, years of professional experience, weekly working hours, number of surgeries performed per week, number of injuries, frequency of pain caused by the injury in the following 6 months, severity of the pain, impact of the injury on immediate surgical performance, impact of the injury, potential interruption of operations, location of the injury, number of sick leave days, existing preventive measures in the workplace, and improved preventive measures implemented after experiencing an injury. Years of professional experience, working hours, and the number of surgeries were recorded in increments of 10. Years of professional experience were grouped into categories of 0–10 years, 11–20 years, 21–30 years, and over 30 years. Working hours were grouped into categories of 20–39 h, 40–59 h, 60–79 h, and > 80 h. The number of surgeries was divided into groups of 0–10 surgeries, 10–20 surgeries, and > 20 surgeries per week. The number of injuries allowed for selection between 0, 1, 2–4, and > 4 injuries.

Participants were invited to complete an anonymous, web-based questionnaire to illustrate musculoskeletal injuries and their impact. To minimize statistical bias, a definition of an “occupational injury” was added to the questionnaire. All injuries that occurred during the operation, without the detection of pre-existing conditions, were included. To ensure maximum response rate, the same procedure was repeated on October, 16th, 2022. Both distributions emphasized that there was no compensation for participating in the survey.

For statistical analysis, all questionnaire responses were collected in Excel spreadsheets to provide an overview of the overall distribution of responses. Subsequently, the results were independently evaluated by two different individuals to avoid statistical errors. The software SPSS Statistics 20 (IBM, Armonk, NY) was used for data analysis. Descriptive and explorative analysis was performed. Chi-squared test for comparison of categorical parameters, *t* test for comparison of continuous normally distributed parameters, and Pearson’s correlation coefficient for calculation of correlations was used. A two-sided *p* value < 0.05 was considered to be statistically significant. Furthermore, a graphics software was utilized to graphically display and compare individual groups of responses.

## Results

The questionnaire was distributed to 1122 orthopedic and trauma surgeons and 135 of those responded (12%). The median age of the respondents was the group of 21–30 years of work experience, and the majority 81 (60%) worked in a private practice, while just 1 (0.7%) reported working in private hospital. Twenty-two doctors (16%) indicated their employment at a university hospital, thirty (22%) at a central hospital, and forty-four doctors (33%) reported their work location at a peripheral hospital.

We observed the presence of at least 1 injury in 72 (52%) of the surveyed surgeons, wherein 26 (19%) reported a single injury, 43 (32%) reported 2 to 4 injuries, and 3 (2%) reported more than 4 injuries. This data is illustrated in Table 1.

**Table 1:** Data from the questionnaire regarding occupational injuries of 135 orthopedic and trauma surgeons from Austria regarding work place, professional experience, number of injuries, and locations

Work place	
Private practice	81
Peripheral hospital	44
Central hospital	30
University hospital	22
Private hospital	1
Professional experience	
0–10 years	25
11–20 years	23
21–30 years	49
> 30 years	38
Numbers of injuries	
0 injuries	63
1 injurie	26
2–4 injuries	43
> 5 injuries	3
Location of injuries	
Hand	34
Finger(s)	29
Feet	16
Head	4
Abdomen	3
Neck	2
Other regions	11

### Location of injuries

Among the total of 72 surgeons identified with injuries, the indicated injury locations were as follows, allowing for multiple responses:

The hand was reported as the site of injury by 34 respondents (47%), while 29 (40%) mentioned injuries to their fingers. Foot and ankle was affected in 16 who (22%) reported injuries, and 4 (6%) noted injuries in the head region. In addition, 3 (4%) reported injuries to the abdomen, 2 (3%) to the neck area, and 11 (15%) specified injuries occurring in other regions. This is illustrated in Table 2.

**Table 2:** Web-based questionnaire sent to all orthopedic and trauma surgeons in Austria

Workplace	
	• University hospital
	• Central hospital
	• Peripheral hospital
	• Private practice
	• Other
Specialization	
	• General medicine
	• Lower extremity
	• Pediatrics
	• Sports orthopedics
	• Tumor orthopedics
	• Upper extremity
	• Joint replacement (endoprosthesis)
	• Spine
	• Traumatology
	• Other
Years of professional experience	
	• 0–10
	• 11–20
	• 21–30
	• > 30
Working hours/week	
	• 20–39
	• 40–59
	• 60–79
	• > 80
Number of surgeries/week	
	• 0–10
	• 10–20
	• > 20
Number of injuries	
	• 0
	• 1
	• 2–4
	• > 5
Frequency of pain in the last 6 months	
	• No pain
	• Several times a day
	• Once a day
	• Several times a week
	• Weekly
	• Monthly
	• Not applicable
	• Other
Severity of pain (0–100)	
	• 0–20
	• 21–40
	• 41–60
	• 61–80
	• 81–100
	• Not applicable
Impact on performance during immediate operations	
	• No impact
	• No impact, but mild pain
	• Minor impact
	• Moderate impact
	• Significant impact
	• Not applicable
Was the surgery interrupted?	
	• Yes
	• No
	• Not applicable
Impact of the injury	
	• None
	• Injury reported to healthcare facility
	• Medical first aid required
	• No suitable resources available at healthcare facility
	• Moderate to severe impact on work morale
	• Moderate to severe impact on family life
	• Sick leave for at least 1 day
	• Not applicable
	• Other
Location of injury	
	• Hand
	• Finger
	• Foot
	• Spine
	• Head
	• Neck
	• Abdomen
	• Other region/s: __________
	• Not applicable
Sick leave days	
	• 0
	• 1
	• 2
	• 3
	• 4
	• 5
	• 6
	• 7
	• 1–2 weeks
	• 3–4 weeks
	• 5–6 weeks
	• > 6 weeks
	• Not applicable
Were preventive measures taken at the previous workplace to minimize the risk of injury?	
	• Yes
	• No
	• Not applicable
Have existing preventive measures been optimized after the injury?	
	• Yes
	• No
	• Not applicable

### Practice setting

When comparing the frequency of injuries between public hospitals and private doctors, the difference was 45% versus 51% without statistically significant difference.

### Age

The questionnaire offered age categories in 10-year increments, ranging from 20–30 years to older than 60. The group of respondents aged 11–20 had the highest injury rate at 65%, while the group 0–10 years had the lowest injury rate at 40%.

### Subspecialty

**Fig. 1:**
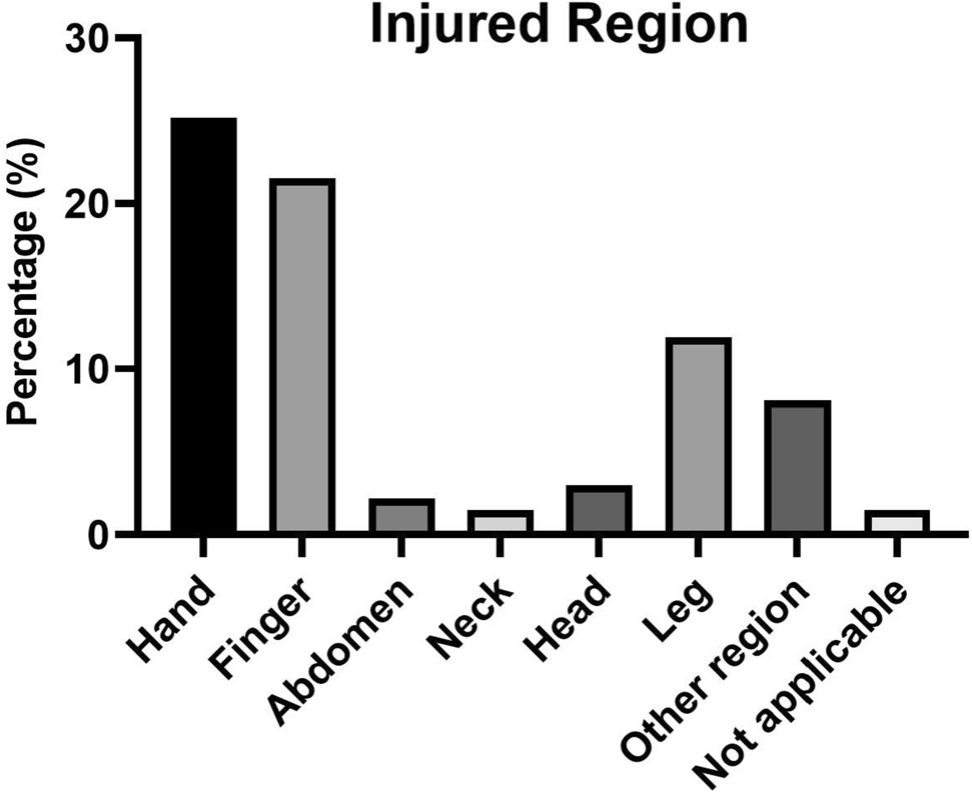
Percentage of detected injury regions among the total of 72 out of 135 surgeons who reported at least 1 injury region

**Fig. 2:**
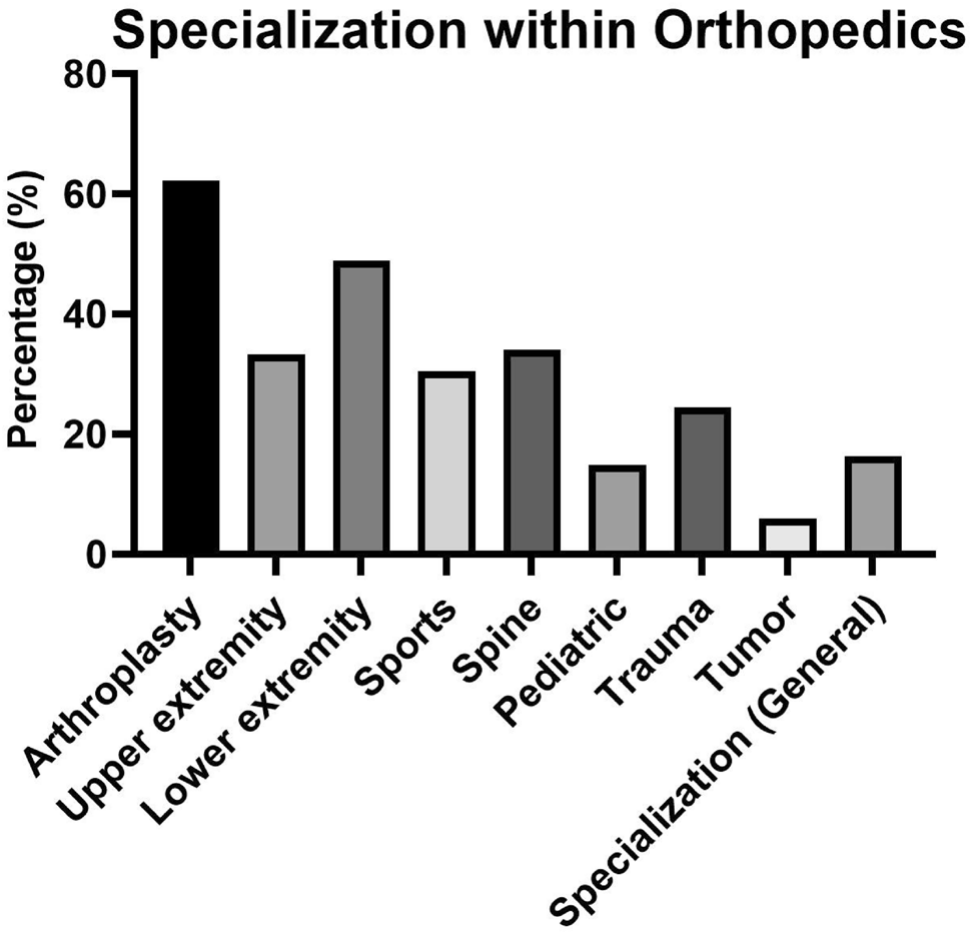
Percentage of detected specialization areas of the 135 surveyed surgeons, with the option for multiple selections

**Fig. 3:**
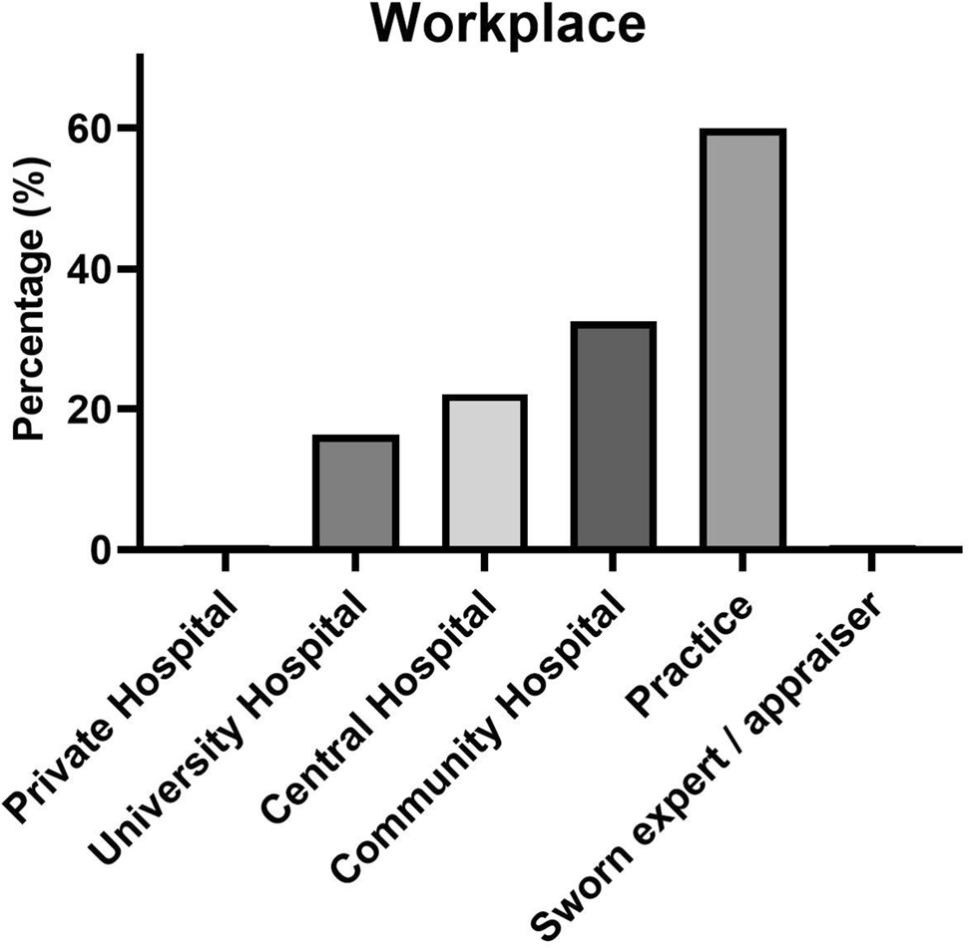
Percentage of detected workplaces among the 135 surveyed surgeons, with the possibility of multiple selections

All subspecialties of orthopedics and traumatology were included in this study and are represented in Fig. 1. Of the 135 doctors who reported at least one subspecialty, the pediatric group had the highest injury rate at 65%, while the subspeciality tumor revealed the lowest injury rate at 37.5% (Figs. 2 and 3).

### Working hours and years of performing surgery

These parameters were categorized in 10-h/year increments, ranging from 20 to > 80 h per week and from 0 to over 30 years of professional experience. Doctors who had 21–30 years of professional experience reported the highest incidence of work-related injuries, followed by those with more than 30 years of experience, indicating a correlation between professional experience and injury risk. A significant association was found between work hours per week and injury frequency as the group with the lowest work hours per week has just a injury percentage of 40% whereas 77.8% of the surgeons who work between 60 and 79 h/week have already suffered from at least one occupational injury.

### Post-injury responses and chronic pain

Fourteen (10%) of the doctors who reported at least one injury had received initial care after the work-related incident. In addition, five doctors went on sick leave after the injury and were unable to perform their work duties. Seventy-two doctors (53%) reported experiencing long-term effects from the work-related injury up to 6 months after injury, resulting in chronic pain that negatively impacted their ability to work.

## Discussion

The aim of this study was to retrospectively capture the number of occupational injuries among orthopedic and trauma surgeons in Austria, identify factors that negatively influence the risk of injuries, and illustrate the impact of occupational accidents on the Austrian healthcare system.

The hypothesis was that the percentage of occupational injuries among orthopedic and trauma surgeons aligns with the incidence reported in the United States [13], thus indicating the need for a workplace prevention program. Furthermore, it is suggested that the number of work absences significantly diminishes the overall performance within healthcare facilities, in terms of sick leave days.

We detected a percentage of 53% of occupational injuries among surgeons in orthopedics and trauma in Austria, which corresponds to a higher percentage of injuries compared to the comparative study performed in the United States [13]. Furthermore, this value clearly exceeds the average for Austrian workers [6] and is comparable to other surgical disciplines [14–16]. Consequently, orthopedic and trauma surgery is considered a high-risk profession. The high numbers of injuries detected, combined with the general increase in work-related accidents in Austria in recent years, clearly indicate the need for implementing or improving prevention measures in individual medical healthcare institutions. Furthermore, there are several studies on the topic of “occupational injuries” that have examined the incidences and impacts, also asserting that a prevention program is clearly warranted. This study serves solely to emphasize the urgency of such a program [1–3, 5]. However, the implementation itself is not carried out in this study, as it falls within the purview of policymaking.

In consideration of the physically demanding environment of orthopedic and trauma surgery, preventive measures represent a logical and essential step toward enhancing the safety of this profession [17, 18]. However, despite multiple instances of compelling scientific evidence, there is a conspicuous absence of a prevention program in the country under examination [1–3, 5]. One relatively straightforward initiative would involve optimizing the ergonomics during surgical procedures to the greatest extent possible [19]. As demonstrated in a study by Albayrak et al., this can be achieved through the ergonomic refinement of surgical instruments [20]. In addition, studies indicate that engaging in physical activity outside of working hours has a beneficial impact on overall physical well-being and contributes to a reduction in the number of sick days taken [21–23].

While injuries to the hand and fingers, such as needlestick injuries, may be associated with less severe harm, injuries to areas such as the spine or head have also been reported, which are associated with a significant disease course and outcome [18]. Moreover, needlestick injuries pose a noteworthy risk of contracting blood-borne diseases such as HIV or HCV [5, 24]. Controversially, a study revealed that surgeons themselves often underestimate and do not take such injuries seriously enough, thereby exacerbating the problem [25, 26].

According to the survey, the resulting sick leaves and chronic pain have a direct negative impact on the surgeon’s work performance, furthermore leading to financial burden on the Austrian healthcare system. During a sick leave, the surgeon is unable to provide patient treatments, yet they are entitled to sick payment. In the worst-case scenario, such as in the case of disability, the surgeon may be absent from the workplace for an extended period, leading to a long-term reduction or complete loss of performance in the operating room.

As expected, we observed a correlation between years of professional experience and the frequency of injuries. However, the highest value was found among those with 21–30 years of professional experience. Interestingly, the group of the eldest, specifically those over 30 years of experience, showed just the second highest incidence of occupational injuries. This could be attributed due to the fact that older surgeons, due to earlier prevailing safety measures, are less inclined to report injuries.

Given the current shortage of doctors in Austria, particularly surgeons, it is crucial to protect the available workforce from absences to continue delivering adequate care. In addition, the field of orthopedics and trauma should be made attractive to medical school graduates to ensure an adequate supply of personnel for both surgical and conservative orthopedic and trauma care in the future.

As this study is the first of its kind in Austria for this professional field, it is subject to certain limitations. Since the questionnaire was individually answered based on the perceptions of the participants, there might be a certain bias present. Furthermore, on one hand, there could have been an over-reporting of injuries as some participants might have been more inclined to emphasize the topic, while on the other hand, some study participants may have withheld reporting injuries due to their focus on selfless patient care without considering their own health. It should be mentioned that the number of orthopedic members in the ÖGO exceeds the number of traumatologists, so the results are more influenced by orthopedic surgeons.

We want to underline the limitation that only 12% returned the survey and we cannot report on the remaining 88% of surgeons in this field in Austria. However, this number is in line with a previous study performed in the Unites States [13], and we believe that there is a positive selection bias, indicating more injuries in the group of surgeons who returned the survey.

In conclusion, it can be said that we found a high incidence of occupational injuries among orthopedic and trauma surgeons which mirrors the findings of colleagues from the United States. The impact on the health system consists of absenteeism in the workplace of highly specialized health service providers as well as the incapacity to work of a high quality, highly trained workforce of at least 4%. With more preventive measures and more attention and care in the rehabilitation phase after such injuries, a positive effect could be achieved. We believe that residents should be specifically trained on how to avoid such injuries in their professional career.

## Data Availability

Anonymous data of the completed questionnaires of 135 orthopaedic and trauma surgeons are available on request.
